# Effect of Activated Platelet-Rich Plasma on Chondrogenic Differentiation of Rabbit Bone Marrow-Derived Mesenchymal Stem Cells

**DOI:** 10.1155/2021/9947187

**Published:** 2021-08-25

**Authors:** Zhen Wang, Zheng Wang, Bin Zhang, Qinghua Zhao, Yubao Liu, Wei Qi

**Affiliations:** ^1^Department of Orthopedics, The First Hospital Affiliated to China Pharmaceutical University, Nanjing 211500, China; ^2^Department of Orthopedics, Zhenjiang Jinshan Hospital, Zhenjiang 212000, China

## Abstract

We aimed to evaluate the effect of activated platelet-rich plasma (PRP) on proliferation and chondrogenic differentiation of bone marrow-derived mesenchymal stem cells (BMSCs). Six mature male rabbits were included in this study. PRP was obtained by two-step centrifugation from whole blood, and it was activated using CaCl_2_ solution. BMSCs were isolated and proliferated from bone marrow of rabbits and characterized by flow cytometry. Passage 3 BMSCs were cultured in high-glucose Dulbecco's modified Eagle's medium (HG-DMEM) with the four different compositions for consecutive 7 days, including 10% fetal bovine serum, 5% PRP, 10% PRP, and 15% PRP. Cell counting assays were performed to evaluate the cell proliferation of BMSCs. BMSCs (5 × 10^5^ cells/well in 6-well plates) were induced in four conditions for 21 days to chondrogenic differentiation evaluation, including commercial chondrogenic medium (control), 5% PRP (HG-DMEM+5% PRP), 10% PRP (HG-DMEM+10% PRP), and 15% PRP (HG-DMEM+15% PRP). The gene expression levels of ACAN, COL2A1, and SOX9 in pellets were detected. Morphological and pathological assessments were performed by the blind observer. After purifying, the percentages of cells with CD105(+)/CD34(−) and CD44(+)/CD45(−) were 96.5% and 92.9%, respectively. The proliferation of BMSCs was enhanced in all groups, and 10% PRP revealed more significant outcome than the others from day 5. The levels of ACAN, COL2A1, and SOX9 were lower in the three PRP groups than control group, but the levels of ACAN and SOX9 were higher in 10% PRP group than 5% and 15% PRP groups. Histological examinations showed that 10% PRP-treated pellets had more regular appearance, larger size, and abundant extracellular matrix than 5% or 10% PRP groups, but still inferior to commercial chondrogenic medium. In conclusion, our results show that PRP may enhance the proliferation of rabbit BMSCs. However, PRP have limited effect on chondrogenic differentiation in comparison with commercial chondrogenic medium in pellets culture.

## 1. Introduction

Clinically, repairing massive articular cartilage defect remains a challenging issue. Current available surgical techniques include osteochondral transplantation and autologous chondrocyte implantation [1, 2]. Despite of relative satisfying results, these techniques have been limited by morbidity of the donor sites and loss of the chondrogenic phenotype during *in vitro* expansion [3]. Alternatively, whether tissue-engineered cartilage can effectively repair massive articular cartilage defects has been a novel focus in this field.

Compared with chondrocytes, bone marrow-derived mesenchymal stem cells (BMSCs) have the potential for chondrogenic differentiation, self-renewal, and proliferation with less loss of phenotypes, which have been considered as an ideal cell source for tissue-engineered cartilage formation [4]. Previous studies have showed that BMSCs can be induced into hyaline-like cartilage tissue with high expression of aggrecan (ACAN), collagen type II (COL2), and SOX9 in high-density pellets or scaffold *in vitro* culture [5, 6]. However, the extremely low percentage of BMSCs in bone marrow means that *in vitro* expansion is the first step to obtain enough cells before chondrogenic differentiation. Besides, the suitable medium containing growth factors is also an essential factor for tissue engineering. So far, the most widely applied mediums are almost commercially synthesized reagents, which have shown excellent chondrogenic-differentiation capacity in tissue-engineered cartilage formation [6–8]. While these medium contains kinds of ectogenic growth factors and are cost expensive, it is still worthy of further study whether some autologous biomaterials with similar induction effects can be an alternative to the synthetic reagents.

Platelet-rich plasma (PRP) is a plasma separated from autologous whole blood, with a high platelet proportion. After activation, PRP releases large amounts of growth factors, including transforming growth factor-beta (TGF-*β*), platelet-derived growth factor (PDGF), insulin-like growth factor-1 (IGF-1), and vascular endothelial growth factor (VEGF). They can enhance tissue repair and regeneration. Clinically, PRP has been shown to inhibit inflammation, and induce cell proliferation and differentiation, resulting in pain relief and functional improvement in some musculoskeletal regenerated diseases [9–11]. Meanwhile, PRP plays an important role in the proliferation of chondrocytes and mesenchymal stem cells (MSCs) [12–15], and the induced chondrogenic differentiation of MSC *in vitro* culture [16–18]. However, limited studies have been reported on whether there are any differences in chondrogenic differentiation between autologous PRP and commercially synthesized medium, or whether PRP may replace these medium or not.

In the current study, we aimed to evaluate the effects of different concentrations of PRP on the proliferation and chondrogenic differentiation of BMSCs cultured in 3D pellets *in vitro*. We also determined if PRP alone was sufficient to induce the chondrogenesis of BMSCs in comparison with traditional chondrogenic differentiation medium.

## 2. Materials and Methods

### 2.1. Experimental Animals

Six mature male rabbits weighting (1.75 ± 0.25) kg (Qinglong Mountain Experimental Animal Center, Nanjing, China) were used in this study. The animal experiments were approved by the Yangzhou University Medical School Animal Ethics Committee (YZMC/IACUC 2019101801, Yangzhou, China).

### 2.2. PRP Preparation

Under anesthetized condition, 50 ml of whole blood was aspirated via heart puncture from each rabbit using 10 ml vacutainer tubes containing 1 ml sodium citrate (Nigale, Chengdu, China). PRP was prepared by a two-step method in room temperature according to our previous study [19]. Briefly, the first centrifugation was performed at 250 × *g* for 7 min; then, the upper plasma layer and middle buffy coat were gathered into another sterile coring tube to the second centrifugation at 350 × g for 10 min. About 2 ml of sediment and plasma were collected from the bottom to obtain PRP. All products were activated with one-tenth volume of 10% CaCl_2_ solution and incubated overnight at 37°C; then, they were centrifuged at 1000 × *g* for 10 min to collect the supernatant to get activated PRP. The activated PRP samples were stored at -70°C and used within one month for subsequent experiments.

### 2.3. Assay of Growth Factors and Cytokines

One milliliter of activated PRP and plasma of whole blood from each animal were used for the measurement of growth factors and cytokines. The levels of TGF-*β*3, PDGF, IGF-1, and VEGF in samples were detected by enzyme-linked immunosorbent assay kits (Cusabio, Wuhan, China) with commercial kit (Nanjing Jiancheng Bioengineering Research Institute, Nanjing, China) according to manufacturer's instruction.

### 2.4. Isolation and Characterization of BMSCs

About 5 ml bone marrow samples were obtained from each rabbit bilateral iliac crests and washed twice with high-glucose Dulbecco's modified Eagle's medium (HG-DMEM, Gibco, Waltham, MA, USA). They were resuspended in HG-DMEN with 10% (*v*/*v*) fetal bovine serum (FBS) (Gibco, Thermo Fisher Scientific, Waltham, MA, USA), 100 U/ml penicillin, and 0.1 mg/ml streptomycin. After 5 days of culture in an incubator with 5% CO_2_ at 37°C, nonadherent cells were removed; then, the medium was changed. Remaining adherent cells were further cultured until 80% confluence, then passaged at a ratio of 1 : 3 in new flasks. Culture medium was exchanged twice weekly. The phenotype of the passage 3 (P3) cells was characterized by detecting surface expression of CD34-phycoerythrin (PE) (GTX75414), CD45-PE (GTX01462-08), CD44-fluorescein isothiocyanate (FITC) (GTX76381), and CD105-FITC (GTX11415) (Genetex, CA, USA) by flow cytometry according to manufacturer's instructions [20].

### 2.5. Cell Proliferation Assay

To observe cell proliferation in different medium, P3 BMSCs were enzymatically harvested and continued to culture in HG-DMEM + 10% FBS (*v*/*v*), HG-DMEM + 5% PRP (*v*/*v*), HG-DMEM + 10% PRP, and HG-DMEM + 15% PRP, respectively. BMSCs were cultured in 6-well culture plates (5 × 10^3^ cells/100 *μ*l/well), and the medium were changed every 3 days. Cell proliferation was assessed using a cell counting kit-8 (CCK8; Abcam, Cambridge, MA, USA) on days 1, 3, 5, and 7 of culture, as described previously [21].

### 2.6. BMSC Pellet Culture

Chondrogenesis was evaluated using high-density pellet cultures. P3 BMSCs (5 × 10^5^/well) were pelleted in 6-well culture plates by centrifugation at 150 × *g* for 5 min and then cultured in four different groups, including control (Ctrl), commercial chondrogenic medium (Cyagen, Guangzhou, China), 5% PRP (HG-DMEM + 5% PRP (*v*/*v*)), 10% PRP (HG-DMEM + 10% PRP), and 15% PRP (HG-DMEM + 15% PRP). Pellets were cultured for 21 days consecutively, and the culture medium was changed three times a week. Eight replicates were assigned for each group.

### 2.7. Reverse Transcription-Polymerase Chain Reaction (RT-PCR) Analysis

BMSCs were harvested at day 21. From each group, 2 samples were homogenized in cold RIPA buffer (Beyotime Institute of Biotechnology, Shanghai, China), then extracted with total RNA extraction with the RNAiso Plus Kit (Takara, CA, USA). Complementary DNA (cDNA) was synthesized from RNA using the PrimeScript™ RT reagent Kit (Takara, CA, USA). The mRNA expression levels of aggrecan (ACAN), collagen type I (COL2A1), and SOX-9 were detected by RT-PCR, as described previously [22]. The primer sequences were designed and synthesized by General Biol (Hefei, Anhui, China) (Table 1). The products of RT-PCR were quantitatively analyzed by the comparative CT method (^ΔΔCT^), and data were presented as target gene expression normalized to GAPDH (Sangon Biotech Co., Ltd., Shanghai, China) (Table 1).

### 2.8. Morphological Observation and Histological Examination

At the end of 3-week chondrogenic differentiation culture, followed by gross observation, all the pellets from the four groups were harvested and fixed in neutral-buffered formalin overnight at 4°C. They were embedded in paraffin, and 5 *μ*m sections were cut and stained with standard toluidine blue (Bioscience & Technology (Wuhan) Co., Ltd., Wuhan, China) to evaluate the production of extracellular matrix (ECM). Immunohistochemical staining was performed to detect COL2 expression (anti-COL2 antibody, rabbit polyclonal to COL22A1, Abcam, Cambridge, MA, USA; anti-GAPDH antibody, rabbit polyclonal antibody against GAPDH, Sangon Biotech Co., Ltd., Shanghai, China), according to manufacturer's instructions. All the sections were observed by a pathologist blindly. GAPDH was considered as the internal reference.

### 2.9. Statistical Analysis

All data were expressed as means ± standard deviation (SD). Statistical analyses were performed using SPSS 18.0 (SPSS, IBM Software Inc., Armonk, NY, USA). Independent-sample *t*-tests were used to evaluate the differences of growth factor levels in plasma and PRP. One-way analysis of variance (ANOVA) was used in the comparison of cell proliferation and gene expression levels, followed by the LSD test to assess the multiple comparisons. *P* < 0.05 was considered statistically significant.

## 3. Results

### 3.1. Growth Factor Levels

**Figure 1:**
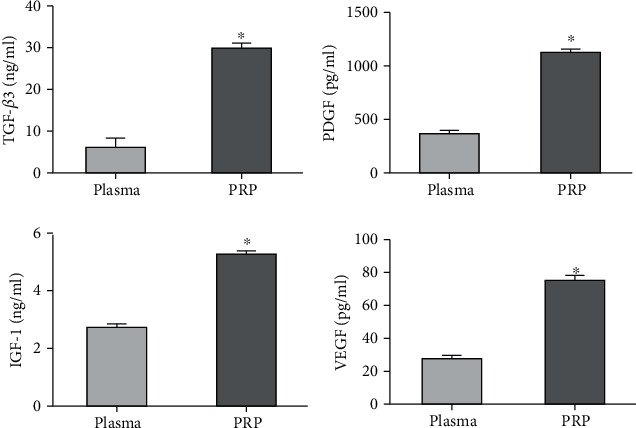
Growth factor levels in plasma and activated PRP. Concentrations of TGF-*β*3, PDGF, IGF-1, and VEGF were significantly increased in PRP compared with plasma (^∗^*P* < 0.05, *n* = 6).

The concentrations of TGF-*β*3, PDGF, IGF-1, and VEGF were significantly increased in activated PRP compared with plasma (TGF-*β*3: 30.49 ± 2.22 vs. 6.62 ± 1.76 ng/ml; PDGF: 1134.00 ± 27.26 vs. 371.42 ± 56.24 pg/ml; IGF-1: 5.24 ± 0.42 vs. 2.72 ± 0.32 ng/ml; and VEGF: 76.22 ± 6.23 vs. 28.10 ± 6.05 pg/ml, respectively, *P* < 0.05, Figure 1).

### 3.2. Characteristics of Isolated BMSCs

**Figure 2:**
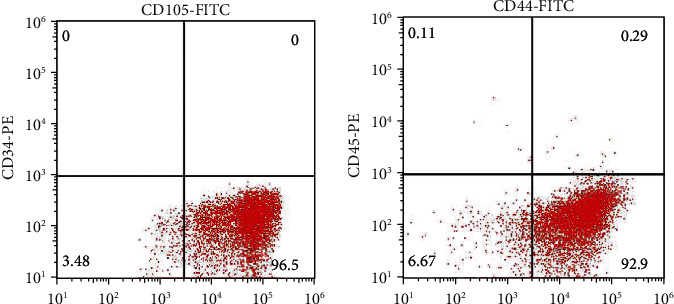
Surface marker profiles of isolated and cultured BMSCs. The percentages of CD105+/CD34 (a) and CD44+/CD45 (b) cells were 96.5% and 92.9%, respectively.

After isolation and culture *in vitro*, P3 cell populations were characterized by flow cytometry to determine the expression of MSC-associated surface markers. The percentages of cells with CD105(+)/CD34(−) and CD44(+)/CD45(−) were 96.5% and 92.9%, respectively (Figure 2).

### 3.3. BMSC Proliferation in PRP

**Figure 3:**
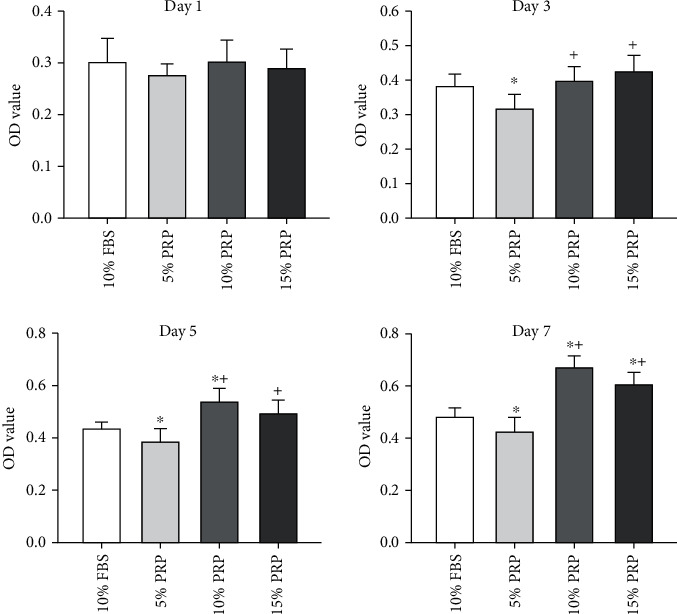
Cell counting assay for BMSC proliferation in the different medium. ^∗^*P* < 0.05 compared to 10% FBS group, ^+^*P* < 0.05 compared to 5% PRP group. OD: optical density; FBS: fetal bovine serum; PRP: platelet-rich plasma.

The cell proliferation BMSCs in 10% FBS, 5% PRP, 10% PRP, and 15% PRP groups after 1, 3, 5, and 7 days of culture were shown in Figure 3. Although proliferation increased with time was observed in all the groups, the effect was significantly weaker in the 5% PRP group compared with the 10% FBS group (*P* < 0.05). BMSC proliferation was enhanced with the increasing PRP concentration, and stronger effects revealed in the 10% and 15% PRP groups compared with the 10% FBS group from day 5 (*P* < 0.05). Furthermore, proliferation assay appeared to be more obvious in 10% PRP group than 15% PRP group from day 5, though no significant difference was observed. Moreover, 7 days *in vitro* amplification results showed that compared with 10% FBS group, the proliferation rate in the 5% PRP group was increased by 0.84 times, that in the 10% PRP group was increased by 1.41 times, and that in the 15% PRP group was increased by 1.27 times (*P* < 0.05).

### 3.4. Expression of Cartilage-Specific Genes

**Figure 4:**
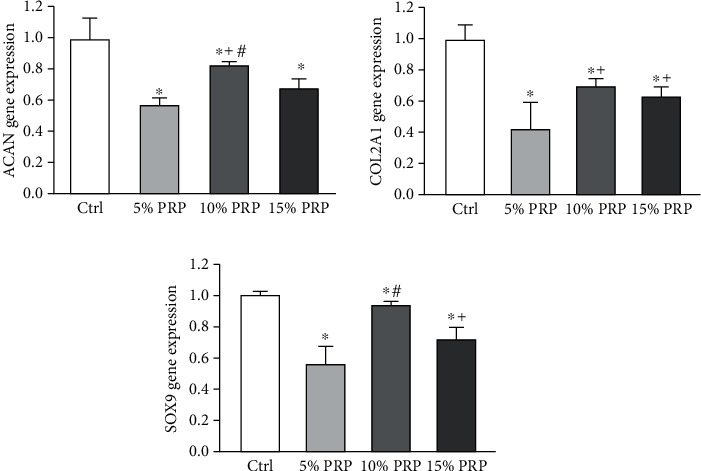
Expression of cartilage-specific genes in the four groups following 21-day culture. Ctrl: commercial chondrogenic differentiation medium. ^∗^*P* < 0.05 compared to control group, ^+^*P* < 0.05 compared to 5% PRP group, ^#^*P* < 0.05 compared to 15% PRP group.

The mRNA expression levels of ACAN, COL2A1, and SOX9 in cell pellets under the four different chondrogenic differentiation conditions after 21 days of culture were shown in Figure 4. The mRNA expression levels of ACAN and SOX9 were significantly higher in the 10% PRP group than the 5% and 15% groups, and COL2A1 mRNA expression was increased in 10% and 15% PRP groups compared with 5% PRP group (*P* < 0.05). However, their mRNA expression levels were all lower in PRP-treated groups compared with control group (*P* < 0.05).

### 3.5. Gross Morphology and Histological Examination

**Figure 5:**
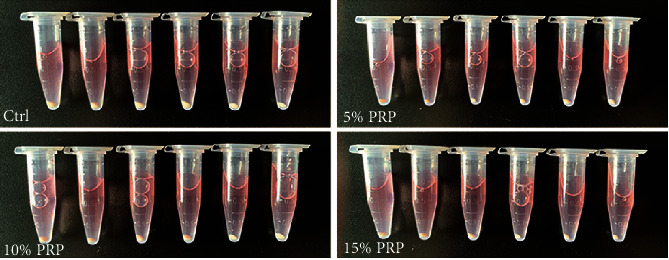
Gross morphology of BMSC pellets in four different media after 3-week culture *in vitro*.

After 3-week *in vitro* culture, all the cell pellets revealed spheroid appearance, but with different sizes (Figure 5). Generally, the pellets in the control group revealed larger volume than that in the PRP-treated groups. However, the pellet sizes did not increase in line with PRP concentration. The diameter of pellets cultured in 15% PRP was similar to 5% PRP, and pellets exposed to 10% PRP were obviously larger than those in 5% and 15% PRP groups, but still less than the control group.

**Figure 6:**
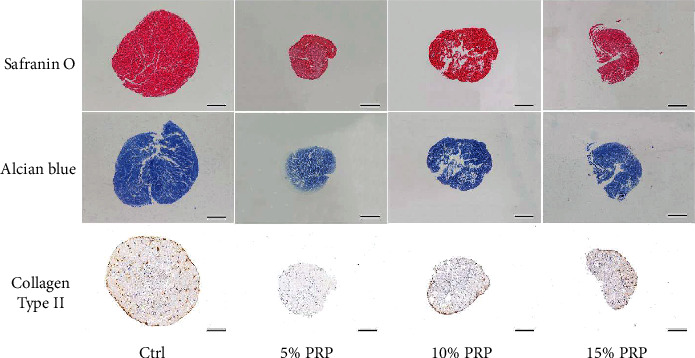
Histological evaluation of aggrecan and collagen type II deposition in BMSC pellets from four different groups after 3-week culture *in vitro*. Scale bar = 500 *μ*m.

**Figure 7:**
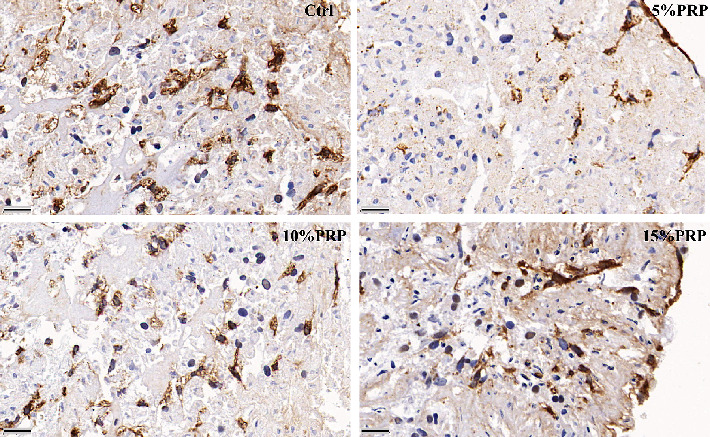
Type II collagen immunohistochemistry of BMSC pellets after 3-week culture in vitro in commercial chondrogenic medium (Ctrl) and 3 PRP groups. Positive staining is indicated by a reddish brown color. Scale bar = 20 *μ*m.

Contents of cartilage-specific ECM, including ACAN and COL2, were staining with Safranin O, Alcian Blue, and immunohistochemistry (Figure 6). More abundant ACAN and COL2 depositions were observed in the control group than PRP-treated groups The pellets exposed to 10% PRP showed stronger ACAN and COL2 staining compared with the other two PRP groups, but still inferior to the control group. The enlarged image of immunohistochemical staining showed that there were more chondrocytes in the control group, and there was COL2 positive staining around them, followed by 10% PRP group and 15% PRP group. The content of chondrocytes and COL2 in 5% PRP group was the least (Figure 7).

## 4. Discussion

PRP is an autologous blood product containing a variety of bioactive components with anti-inflammatory, tissue repair, and regeneration-promoting functions, which has been widely used in the treatment of chronic degenerative diseases [9–11]. *In vitro* experiments have confirmed the ability of PRP to enhance the proliferation of chondrocytes and MSCs [12–15]. However, the effect of PRP on cartilage differentiation of BMSCs in tissue engineering remains controversial. Although recent studies [17, 18, 23] have demonstrated the effects of different concentrations of PRP on chondrogenic differentiation of MSCs, they do not compare PRP with traditional commercial cartilage induction medium. Therefore, we aimed to compare the effects on cartilage differentiation of BMSCs between different PRP concentrations and commercial cartilage induction medium and to evaluate the role of PRP in tissue-engineered cartilage formation.

In this study, the concentrations of TGF-*β*3, IGF-1, PDGF, and VEGF were significantly increased in activated PRP compared with plasma. The addition of high concentrations of PRP to basic medium promoted the expansion of BMSCs *in vitro* compared with 10% FBS, revealing a concentration time-dependent effect. Higher concentrations of growth factors and cytokines in PRP may induce this result.

Commercial MSC chondrogenic differentiation media from different manufacturers have been widely used in chondrogenic differentiation experiments. These synthetic reagents contain not only basic cell culture medium but also additional ingredients, such as TGF-*β*3, insulin, transferrin and selenium supplement, ascorbate, sodium pyruvate, proline, and dexamethasone. Among these, TGF-*β*3 is a vital regulatory factor in promoting chondrogenic differentiation of MSCs, while the other components also improve cell metabolism, promote cell proliferation, and inhibit cell aging. The concentration of TGF-*β*3 in the cartilage differentiation medium used in this study was 10 ng/ml, which was higher than that in 10% PRP (3.05 ng/ml), while PRP itself did not contain any other additives. Therefore, we considered that basic cell culture medium with 10% PRP might have a limited effect on BMSC cartilage differentiation compared with commercial cartilage differentiation medium.

It is not clear that whether the increased PRP concentration in medium would enhance chondrogenic differentiation in BMSCs. Krüger et al. have reported that human PRP enhances the migration and stimulates the chondrogenic differentiation of human BMSCs derived from spongious bone of the tibia or femur head [16]. Liou et al. have investigated the effects of different concentrations of PRP on adipose-derived stem cells, and they have shown that increasing the PRP concentration do not enhance chondrogenic differentiation [18]. Amaral et al. also have observed cartilage differentiation medium containing different PRP concentrations on chondrogenesis of BMSC pellets, and they find that increasing the PRP proportion from 1% to 10% reduces the expression levels of cartilage-specific genes and proteoglycans in pellets [23]. In addition, some previous studies have reported that commercial induction medium containing 10% PRP do not enhance chondrogenic differentiation compared with induction medium alone [17]. The similar results are also observed in this study. Though certain PRP concentration induces chondrogenic differentiation in BMSCs pellets, this effect is not enhanced with the increasing of PRP contents.

In this study, we observed that 10% PRP promoted the chondrogenic differentiation of BMSCs pellets by analysis of gene expression levels, gross morphological observation, and immunohistochemical staining of cartilage-specific proteins. We supposed that the biological mechanism of PRP in chondrogenic differentiation of MSCs may be complicated and intricate. Furthermore, it needs further study to observe whether MSC derived from different sources respond consistently to PRP in induction chondrogenesis.

Activated-PRP includes cytokines, such as TGF*β*, PDGF, VEGF, and epidermal growth factor (EGF), but the beneficial effects of these growth factors in chondrogenesis remain unclear. The TGF*β* family is known to induce proliferation and chondrogenesis of BMSCs during cartilage formation. Meanwhile, PDGF supports chondrocytes to maintain the hyaline-like chondrogenic phenotype and induces proteoglycan synthesis [24]. However, VEGF shows poor chondrogenic effects on muscle-derived stem cells in a rat model [25], and an EGF receptor ligand promotes chondrocyte catabolic activity and inhibits anabolic activity in an osteoarthritis mouse model [26]. VEGF and/or EGF may therefore weaken the chondro-inductive effects of PRP. Further studies are needed to determine if specific depletion of such antichondrogenic factors and optimization of other stimulatory components in PRP might enhance BMSC chondrogenic differentiation.

In addition to optimizing the differentiation induction medium, it is also necessary to improve the seed cells to facilitate the construction of tissue-engineered cartilage. Some researchers have constructed a coculture system composed of autologous chondrocytes and MSCs in order to observe the efficiency of cartilage differentiation and to inhibit chondrocyte hypertrophy and osteogenic differentiation by the paracrine effect of different cells [27, 28]. Although the results were not consistent, they provided a novel research direction for the construction of tissue-engineered cartilage. Recent studies have reported that human placental [29], umbilical cord blood [30], and amnion-derived MSCs [31] reveal high proliferative ability, multipotency, and low immunogenicity, which become novel seed cells in tissue engineering. Further studies are needed to observe whether PRP could enhance chondrogenic differentiation in these stem cells or not.

There are also some limitations in this study. Firstly, we only observed the end-stage chondrogenic differentiation of BMSCs after 21 days of culture, and the continuous effect of PRP were not examined. Secondly, limited number of PRP-treated groups were designed; the details of concentration-dependent effect of PRP on chondrogenesis were not enough. Additionally, due to commercial chondrogenic differentiation media from different manufacturers may contain various components and efficacy, the reagents used in this experiment are of limited representativeness.

## 5. Conclusion

Activated-PRP contains abundant growth factors and cytokines, which can enhance BMSC proliferation *in vitro* and induce chondrogenic differentiation in 3D pellet culture. However, the effects of PRP alone used were limited compared with traditional commercial MSC chondrogenic differentiation media. Future studies that aim at optimizing and modifying the composition and concentration of PRP may have satisfying results in tissue-engineered cartilage formation.

## Figures and Tables

**Table 1:** Sequences of primers for RT-PCR analysis.

Genes	Primer sequences	Product (bp)
ACAN	F-AACAGCCCAAGAAGCAGAA R-TGGGTCCAGAAATCCAGAATG	103
COL2A1	F-CAAGTCCCTCAACAACCAGAT R-TATCCAGTAGTCACCGCTCTT	124
SOX9	F-GGAGGAAGTCGGTGAAGAATG R-TGCAGCGCCTTGAAGAT	91
GAPDH	F-AATCCACTGGCGTCTTCAC R-TCACGCCCATCACAAACA	117

## Data Availability

The datasets used in the current study are available from the corresponding author on reasonable request by email.
